# Building CRESCENDO – a global registry of glomerulonephritis with crescents

**DOI:** 10.1093/ckj/sfag077

**Published:** 2026-03-05

**Authors:** Vanja Ivković, Andreas Kronbichler, Annette Bruchfeld, CRESCENDO Investigators, D Adu, F Aqeel, F Alberici, S Almaani, U Anandh, M Arnol, F Arogundade, I Bajema, J Barratt, P Batkovi?, S Bell, A von Bergwelt-Baildon, J Boavida, J Jacques Boffa, P Boor, B Bose, O Boyer, B Brilland, S R Brix, I Bubi?, S Bulimbašić, D Buob, M Busch, P Javier Cannata Ortiz, F Caravaca-Fontan, G Cavri?, T Caza, A Chalkia, A Chellappan, C Kay Cheung, R Chorao, A Maria Cordoba Huerto, M ?ori?, S Correia, Z Cui, A Dhaygude, E Esteve, S Faguer, R Fernandez Prado, M Fischereder, J Floege, L Floyd, L Francis, E Frangou, E Fu, S Fujimoto, D P. Gale, K Gale�i?, D Gale�i? Ljubanovi?, J Gameiro, L Cordero Garcia Galan, D Geetha, L Gesualdo, I Godinho, M Gois, A Gomes, D Goumenos, M Hassan, M Haubitz, M Hilhorst, I Horvati?, Z Hruskova, T B. Huber, J Il Shin, A Inacio, W Ismail, T Ito-Ihara, A Jauhal, D Jayne, R Jones, A Jonsson, R Kalyesubula, T Kanjanabuch, D Kasumovi?, N Kojc, A Karras, M Kikuchi, T Kne�evi?, T Knoop, M Kollar, T Komiya, S Kotwal, V Kr�tk�, V L'Imperio, M Laganovi?, B Lange-Sperandio, I Laranjinha, A Levin, S Lundberg, V Luyckx, G Maalouf, M Maeske, B Maksimovi?, A Malvar, J Malyszko, S Marques, B Marques da Silva, M Mbengue, S McAdoo, D Medika Hertanto, J M Mejia Vilet, L Mendonca, S Mirioglu, H Miyata, J Morelle, A Morris, R Muhindo, R Nada, D Nakagomi, K Emil Nelveg-Kristensen, B Neuen, A Niang, E Nogueira, S Norouzi, B Odler, S Ohlsson, I Okpechi, J Oliveira, B A. Omotoso, L Oni, A Ortiz, B Osmani, S Ozturk, M Papasotiriou, E Pardo, S Parikh, C Au Peh, A Julie Peired, M Pereira, N Piko, M Praga, D Pramudya, C Rafat, R Ramachandran, M Rathi, T Rauen, H Reich, I Roberts, K Rohner, A Rosenberg, B Rovin, B Runesson, A Rymarz, D Saadoun, A Saeed, M Sanaei Nurmi, B Sanchez-Alamo, J Sanchez-Rodriguez, J Sanders, S Santos, U Sch�nermarck, H Seeger, M Segelmark, C Seikrit, H Selvaskandan, T Shehab, M Jose Soler, I Soveri, S Steiger, K Stevens, M K. Svensson, P �enjug, M Tedesco, V Tesar, N M. Tomas, S Troyanov, L Uchida, A Vaglio, G Vajgel, G Vasquez-Rios, Željka Večerić Haler, C A te Velde-Keyzer, M Vieira Goncalves, A Wang, M Weiner, M A. C. Wester Trejo, E Westergren, J Wijkstrom, N Zagorec, M Zhao

**Affiliations:** Department of Health, Medicine and Caring Sciences, Linköping University, Linköping, Sweden; Department of Health, Medicine and Caring Sciences, Linköping University, Linköping, Sweden; Department of Internal Medicine IV, Nephrology and Hypertension, Medical University Innsbruck, Innsbruck, Austria; Department of Health, Medicine and Caring Sciences, Linköping University, Linköping, Sweden; Department of Renal Medicine, Karolinska University Hospital and CLINTEC Karolinska Institutet, Stockholm, Sweden

**Keywords:** crescentic glomerulonephritis, crescents, histology, outcome, rapidly progressive glomerulonephritis

## Abstract

Crescents represent a non-specific mechanism in response to severe glomerular injury and show a temporal pattern evolving from cellular and fibrocellular to fibrous. Crescentic glomerulonephritis (GN) is defined as ≥50% cellular crescentic glomeruli on kidney histology and is frequently characterized by rapid deterioration of kidney function and features of nephritis. However, little is known about how different types and proportions of crescents affect prognosis and this may differ across diseases forming the spectrum of crescentic GN. To explore this, we devised CRESCENDO, a multinational, longitudinal, observational registry collecting clinical, laboratory and histopathology data from patients with GN and crescents. The registry aims to enrol patients from all continents to explore the prognostic value of crescents on a continuum, ranging from one crescentic glomerulus to 100% crescents on biopsy. We also aim to explore other potential prognostic and diagnostic predictors of a wide range of outcomes (not limited to mortality and kidney survival, but also infections, malignancy and kidney function recovery), leverage exploratory machine learning approaches of scanned kidney biopsies and test the efficacy and safety of different therapies across the crescentic GN spectrum. The findings of CRESCENDO will hopefully help close gaps in the understanding of crescentic GN and will generate further hypotheses providing the basis for subsequent research.

KEY LEARNING POINTS **What was known:**

- Crescents are a non-specific response to glomerular injury and their presence on kidney histology is associated with worse outcomes.
- Crescents are predictors of prognosis in several glomerular diseases, with cellular crescents indicating disease activity or severity and fibrous crescents being associated with poor kidney outcomes and progression to chronic kidney disease. The role of fibrocellular crescents is less known, but they have also been shown to be associated with disease activity.

**This study adds:**

- We plan to establish the largest global registry of crescentic glomerulonephritis (GN), CRESCENDO, to explore the impact of crescents on outcomes, including the use of deep learning algorithms for diagnostic and prognostic purposes.
- Findings from CRESCENDO may help in understanding the significance of crescents over a continuum and potentially in redefining the ‘classical’ thresholds of crescent percentage for diagnosis and prognosis across the spectrum of crescentic GN.
- With data from CRESCENDO we will evaluate the efficacy and safety of different therapies used in crescentic GN using a target trial emulation approach that provides a setting as close as possible to that of randomized controlled trials.
- We plan to explore a wide range of outcomes, including mortality, kidney survival, kidney function recovery, infections and malignancy.

**Potential impact:**

- Findings from CRESCENDO will contribute to the overall knowledge of histology, predictors (i.e. diagnosis, risk factors for outcomes) and prognosis of GN with crescents.
- Our approach using machine learning and target trial emulation might provide highly reliable results that could impact future recommendations in this field.
- Substudies from our initiative might help generate further hypotheses providing the basis for subsequent research.

## INTRODUCTION

Formation of crescents, deriving from epithelial cells of the Bowman’s capsule, is a non-specific mechanism in response to severe glomerular injury [1]. Rupture of the glomerular basement membrane (GBM) due to inflammation, immune-mediated glomerular injury and invasion of inflammatory cells, subsequent activation of the complement system, neutrophil extracellular traps (NETs) formation and deposition of coagulation factors into Bowman’s space promote further injury [2]. Crescents differ in composition and evolve from an intra- and extracapillary fibrinoid necrosis that advances to crescents and fibroses over time from a cellular to a fibrocellular and fibrous crescent, depending on the relationship between the proportion of cells and fibrous matrix [1, 3–7]. Fibrous crescents (with >75% fibrous matrix) mark a stage in this evolution associated with chronic changes that correlate with the lowest potential for glomerular filtration rate (GFR) recovery in glomerulonephritis (GN) patients [1].

Crescentic GN (cGN) is defined as at least 50% of crescentic glomeruli on kidney histology and typically manifests as rapidly progressive GN (RPGN), a clinical entity commonly marked by rapid deterioration of kidney function, haematuria and proteinuria in the presence of glomerular crescents on kidney biopsy [8, 9]. However, far less is known on how different types and proportions of crescents can affect prognosis, including reversibility of kidney function loss, as this may differ across diseases forming the spectrum of cGN. To better delineate and understand the phenomenon of crescents and their impact on prognosis, as well as to explore the efficacy and safety of different therapeutic regimens, we wish to establish an international registry of cGN. This registry should also include patients with a lower proportion of crescents on histology (below the 50% threshold) to enable us to fully appreciate the impact of crescents on a continuum, as previous studies have indicated that there is probably no universal threshold and that the association of percentage of crescents and kidney outcomes might be continuous and linear [10]. We have thus devised CRESCENDO, a global registry of cGN, with the aim to close these gaps in knowledge.

### Establishment of CRESCENDO

CRESCENDO is a multinational, longitudinal, observational registry comprising clinical, laboratory and histopathology data from patients with GN and crescents on histology, linked with the European Renal Association (ERA) Immunonephrology Working Group (Table 1). Our aim is to establish a registry of patients from all continents and investigate the roles of crescents in GN, including rare immune-mediated kidney diseases. We plan to include patients with crescents on histology, regardless of aetiology or diagnosis, to explore histological phenotypes and potential factors influencing presentation and outcomes beyond the underlying disease. There will be no minimum number of patients recruited per centre. However, we plan to enrich the registry with very rare forms of GN with crescents, as our registry represents a platform to study these diseases. Thus we will ensure that patients with diseases such as cryoglobulinemia, C3 glomerulopathy and fibrillary GN presenting with crescents are captured. There are several secondary aims related to exploring potential prognostic and diagnostic predictors of a wide range of outcomes, including leveraging exploratory machine learning approaches of scanned kidney biopsies to test its applicability across the spectrum of cGN in linking predictors and outcomes (Fig. 1). The efficacy and safety of different therapies will be assessed using a target trial emulation (TTE) approach enabling a causal inference framework to answer questions of interest. Coupled with broad, high-granularity registry data, this will enable us to account for many types of biases and confounding, leading to high internal validity. Studies using TTE were previously shown to produce results comparable in reliability to those reported in randomized controlled trials (RCTs) [11].

**Figure 1:**
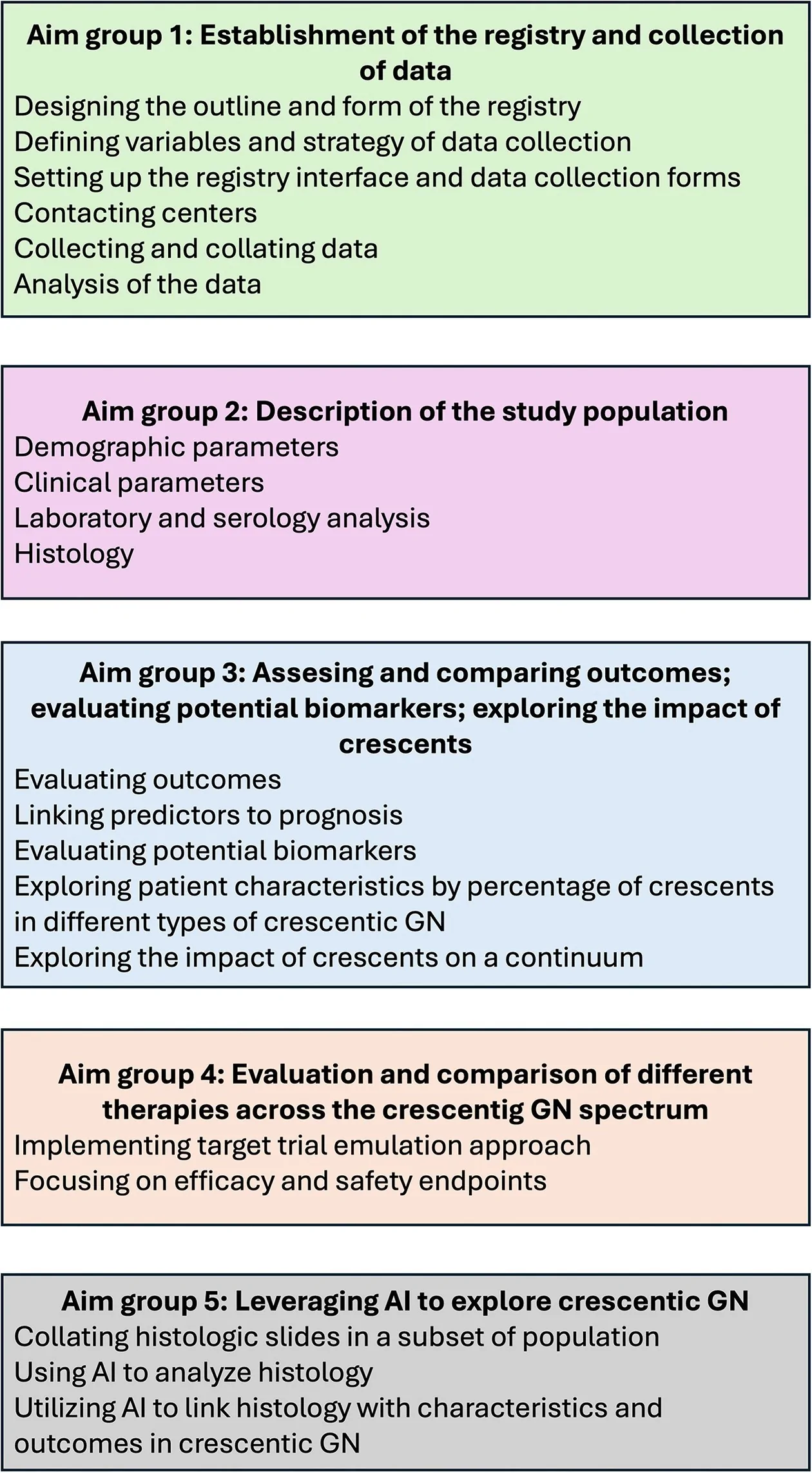
Aims of CRESCENDO grouped in distinct aim groups. Each aim group consists of logically and logistically related aims.

**Table 1:** Main data collected in CRESCENDO.

Section	Data
Background	Socio-economic and anthropometric data, physical examination, medical history
GN and organ involvement	Family history of kidney disease, date of biopsy, date of GN diagnosis, concurrent GN, dialysis dependence, extent of organ involvement at diagnosis, disease activity and damage scores, **histological images** and kidney biopsy report
Laboratory values	Full blood count, **erythrocyte sedimentation rate**, serum creatinine, cystatin C, eGFR, C-reactive protein, serum albumin, **serum immunoglobulins and free light chains**, serology (C3, C4, C1q, ANA, anti-dsDNA, ANCA, **MPO- and PR3-ANCA**, anti-GBM antibodies, **anti-PLA2-R, anti-phospholipid antibodies**), **cryoglobulins, virus serology (hepatitis B, hepatitis C, HIV, other viral diseases)** and urinalysis (24-hour proteinuria and albuminuria, protein:creatinine ratio, albumin:creatinine ratio, dipstick proteinuria and haematuria, **dysmorphic erythrocyturia** and **red blood cell casts**)
GN scoring form	Light microscopy: total number of glomeruli; total number of glomeruli with crescents; crescent extent (segmental or circumferential); number of glomeruli with cellular, fibrocellular and fibrous crescents; number of sclerotic glomeruli; membranoproliferative pattern; endocapillary proliferation and necrosis; IFTA; thrombotic microangiopathy
	Immunofluorescence/immunohistochemistry: intensity (0–3+) and pattern (granular or linear) of IgG, IgM, IgA, **kappa and lambda light chain staining**, intensity of C3 and C1q
	**Electron microscopy: deposits (mesangial/paramesangial, subendothelial, subepithelial), dense deposits, podocyte effacement (focal or global, defined as <50% and ≥50%, respectively), dense deposits, tubuloreticular inclusion**
Non-immunosuppressive therapy	Hypertension therapy (ACEI, ARB and other classes), diabetes therapy (metformin, GLP-1 agonists, insulin), other therapy (SGLT2i, statin, sparsentan, ET1 inhibitors, MRA), non-pharmacological therapy (tonsillectomy, weight loss, cessation of smoking)
Immunosuppressive therapy and PLEX	Immunosuppressive therapy: rituximab, oral and intravenous cyclophosphamide, MMF, intravenous and oral glucocorticoids, CNI (cyclosporine and tacrolimus), methotrexate, azathioprine, avacopan, hydroxychloroquine, obinutuzumab, vocloscporin and belimumab
	PLEX: date of first PLEX, regimen (daily, every other day, twice weekly and other) and number of PLEX procedures, immunoadsorption
New-onset comorbidities at follow-up	Diabetes, hypertension, atrial fibrillation and heart failure

Variables shown in bold represent elements of the expanded dataset, while all remaining variables make up the minimal dataset.

ACEI: angiotensin-converting enzyme inhibitor; ANA: antinuclear antibody; anti-PLA2-R: anti-phospholipase 2 receptor; ARB: angiotensin II receptor blocker: CNI: calcineurin inhibitor; GLP-1: glucagon-like peptide-1; ET-1: endothelin 1; HIV: human immunodeficiency virus; IFTA: interstitial fibrosis and tubular atrophy; Ig: immunoglobulin; MMF: mycophenolate mofetil; MPO: myeloperoxidase; MRA: mineralocorticoid receptor antagonist; PLEX: plasma exchange; PR3: proteinase-3; SGLT2: sodium–glucose co-transporter 2.

Adult and paediatric patients will be included in both a retrospective and prospective longitudinal study, including those who participate or have participated in RCTs (Fig. 2). There is no minimum follow-up time for enrolment, in order to avoid excluding patients with more severe disease who may experience higher mortality rates in the initial months of follow-up. Inclusion criteria will be broad, with the primary criterion being the presence of at least one crescentic glomerulus on kidney biopsy, which will allow for the evaluation of the impact of crescents as a continuum (Fig. 2). Crescents will be defined as per consensus definition for glomerular lesions by the International Society of Nephrology (ISN)/Renal Pathology Society (RPS) revision of classification criteria for lupus nephritis (LN) [1, 12]. Definitions from these classifications that are used in this study are presented in Table 2. Pseudocrescents will not be considered in the total number of crescents [12]. Patients with fewer than five glomeruli on a biopsy sample will be excluded. The threshold was chosen to be in line with the findings of Bjørneklett *et al*. [13], which showed that a minimum of three glomeruli is sufficient. No difference was found in the applicability and predictive value of the Berden classification in biopsies with 3–9 glomeruli compared with those with ≥10 glomeruli. These findings were in line with other large studies using a threshold of a minimum of five glomeruli, such as the ANCA Renal Risk Score and others [14–16]. Other main inclusion and exclusion criteria are presented in Fig. 2.

**Figure 2:**
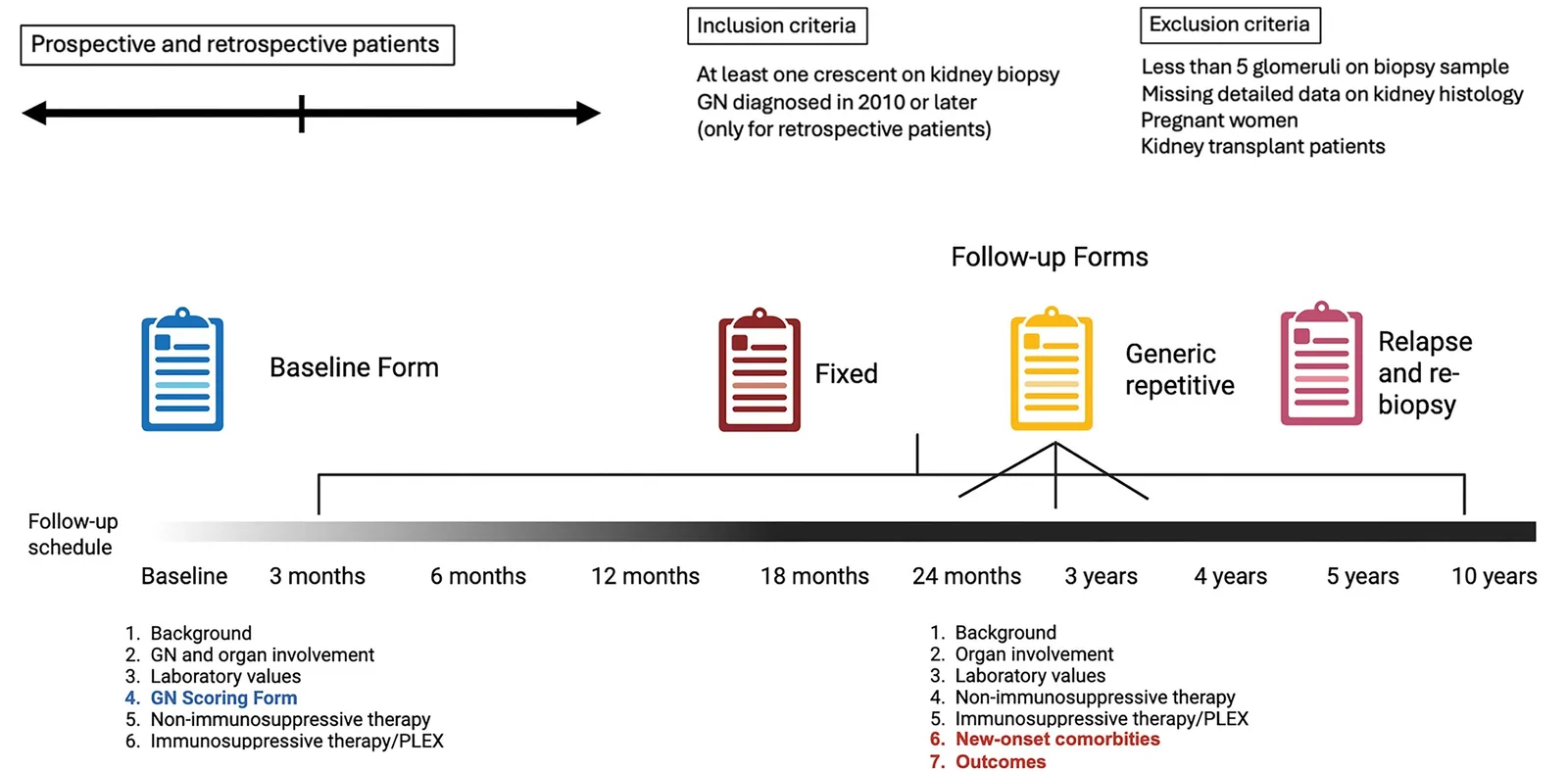
Flow chart of CRESCENDO detailing inclusion and exclusion criteria as well as the follow-up schedule, different electronic case report forms and their sections, which are designed to optimally group, collect and collate data. Data for each patient will be entered at baseline using the baseline form and at each fixed-time follow-up using the fixed follow-up form (up to nine time points per patient: at 3 months, 6 months, 12 months, 18 months, 24 months, 3 years, 5 years and 10 years). Investigators can also enter data at any other time point when an event of interest (as deemed by the investigator herself/himself) has occurred using the generic repetitive follow-up form or at the time of relapse or rebiopsy using the relapse and re-biopsy form. We have marked the GN scoring form (blue) to emphasize that it only appears in the baseline form and the new-onset comorbidities (red) as it only appears in the follow-up form. The relapse or rebiopsy follow-up form will also include the ability to enter data on histology if a rebiopsy was performed.

**Table 2:** Definitions of crescentic lesions according to the Renal Pathology Society 2020 consensus definitions for glomerular lesions by light and electron microscopy and the 2018 revision of the International Society of Nephrology/Renal Pathology Society classification for LN.

Lesion	Definition
Crescent	A lesion consisting of extracapillary hypercellularity, composed of a variable mixture of cells. Fibrin and fibrous matrix may be present; ≥10% of the circumference of Bowman’s capsule should be involved
Cellular crescent	Extracapillary hypercellularity of two or more cell layers and involving >10% of the capsular circumference, composed of >75% cells with or without fibrin and <25% fibrous matrix
Fibrocellular crescent	As cellular crescent, but composed of 25–75% cells with or without fibrin, and the remainder fibrous matrix
Fibrous crescent	Extracapillary fibrosis composed of >75% matrix and <25% cells with or without fibrin and involving >10% of the capsular circumference
Pseudocrescent	Visceral epithelial cell hyperplasia with crowding of these cells filling the urinary space, lacking inflammatory cells and fibrin; the cells are typically plump (not spindle-shaped) and often vacuolated, and Bowman’s capsule is usually intact

The scope of collected data is detailed in Table 3. To summarize, we will collect a wide array of data starting with demographics, patient and family history and physical examination. Furthermore, we will collect data on the initial diagnosis of GN, including data on when the biopsy was performed, disease activity and damage using the most appropriate scoring systems specific to each GN and on extrarenal involvement at diagnosis and each follow-up, such as on diffuse alveolar haemorrhage and involvement of other organs.

**Table 3:** Definition of primary and secondary endpoints.

Endpoint	Definition
Primary endpoint	Time to a composite of kidney failure (defined as an eGFR <15 ml/min/1.73 m^2^, initiation of dialysis or kidney transplantation) and all-cause mortality
Key secondary endpoints	The time to major events (kidney failure, all-cause mortality, 50% decline in eGFR, cardiovascular mortality)
	Kidney failure
	All-cause mortality
	30% and 50% decline in eGFR
	Combinations of kidney failure, mortality and decline in eGFR
	Cardiovascular outcomes: MACE (myocardial infarction, haemorrhagic stroke, ischaemic stroke), cardiovascular mortality
	Annual change in eGFR
	Recovery from kidney replacement therapy
	Severe infections (frequency, type, infection-related death)
	Cancer (skin, other), cancer-related mortality
Other secondary and explorative endpoints	Patient-reported outcomes (quality of life measured with the WHQQOL-BREF and health-related quality of life measured with the SF-36 or equivalent, such as RAND-36)^a^
Safety endpoints	Adverse and serious adverse events with at least a possible relationship to treatment

^a^Patient-reported outcomes will only be assessed in prospectively enrolled patients.

MACE: major adverse cardiovascular events; SAE: serious adverse event; SF-36: 36-item Short Form Health Survey; WHOQOL-BREF: World Health Organization Quality of Life (Brief Form).

The electronic case report form (eCRF) will include a GN scoring form (Fig. 2) in which most pertinent histologic data, such as the number of glomeruli, percentage of each type of crescents and extent of interstitial fibrosis and tubular atrophy will be collected. This section will also collect data as provided by centres on immunofluorescence/immunohistochemistry (staining for immunoglobulins, C3, C1q and light chains) and electron microscopy (podocyte effacement and deposits). This section will allow investigators to input histology reports and, most importantly, to upload whole slide images or other relevant histologic images. Investigators are encouraged to submit all available images for a patient. The latter part is not mandatory but is strongly preferred, whenever possible, as these images will form an important part of the study employing machine learning to differentiate among histologic presentations across glomerular diseases and link histologic hallmarks to outcomes. We plan to also offer central scanning in cases where local scanning is not possible. In these cases, investigators will be asked to send glass slides or other suitable material to the central pathology team. The central pathology team will have access to all histology data and images and perform scanning.

Granular data on immunosuppressive and non-immunosuppressive therapy will be collected at each follow-up (Table 1). We will collect data on interventions and any clinically relevant change in treatment regimens as deemed important by investigators. Finally, we will aim to capture data on new-onset comorbidities such as diabetes and heart failure and on outcomes such as death, kidney failure with kidney replacement therapy (KFRT), transplantation and cardiovascular diseases, but also severe infections, new-onset malignancy, recovery from KFRT, other adverse events and patient-reported outcomes (Table 3).

We will collect all repeat biopsy data as available. Patients who have persistence of a percentage or resolution of all crescents on a repeat biopsy will provide insights into effects of therapies on kidney histomorphology and subsequently relevant clinical outcomes such as recovery from dialysis. CRESCENDO provides a unique opportunity to study predictors of refractory disease and relapse and aims to explore which parameters correlate with the development of crescents in these patients. These longitudinal data will be analysed using repeated-measurements tests to evaluate if there is any association of change in parameters with development of crescents.

Data will be captured using tailored eCRF via Research Electronic Data Capture (REDCap) based at Linköping University, Sweden. Investigators will input data into the eCRF and have access to the data from their own patients. We will form working groups for different areas of research interest that will propose and conduct substudies in their field of interest. Individual investigators or groups of investigators will be encouraged to freely propose ideas for further research as well as dedicated substudies on different research topics that will be reviewed and approved by the Core Committee.

Two types of eCRF will be used: a baseline form, which will include a GN scoring form (Fig. 2), and a follow-up form. To achieve high data granularity and as robust data as possible, there will be three types of follow-up forms: a fixed follow-up form, of which there will be one for each prespecified, fixed follow-up point; a generic repetitive follow-up form, of which there is no prespecified maximum number and in which investigators can enter data at any time in case of any events they consider important (Fig. 2); and a relapse and/or rebiopsy form, which will be used to most efficiently capture data on any potential relapse or other reason for biopsy.

The primary endpoint in CRESCENDO is time to a composite of kidney failure and death. All endpoints are presented in Table 3.

Data analysis will be done using validated statistical tests, as warranted by each specific study using data from the registry. We will aim to construct robust multivariable models that should account for confounding and assess independent associations of predictors and outcomes. Given the multiple aims, necessary sample size calculation cannot be performed at this time but will be performed for each study to evaluate if the study has sufficient power to detect the outcomes.

Ethical approval will first be obtained at Linköping University for Sweden, after which all participating centres will obtain local ethical approvals, if this is required.

## DISCUSSION

A total of 102 centres from 41 countries from all continents, except Antarctica, have so far expressed interest in participating in our initiative. Few studies have focused specifically on crescents as the pathological hallmark of RPGN, evaluating and comparing their impact and effect on prognosis across different GNs. Prior findings suggest that not all crescents are created equal and that different types and proportions of crescents may signal distinct clinical trajectories and outcomes, as seen in conditions such as anti-neutrophil cytoplasmic antibody (ANCA)-associated vasculitis (AAV) compared with anti-GBM disease. In a study of 145 patients with ANCA-GN, 37 (25.5%) patients belonged to the crescentic class. They presented with an estimated glomerular filtration rate (eGFR) of 18 ± 16 ml/min/1.73 m^2^, which increased to 37 ± 21 ml/min/1.73 m^2^ at 12 months [15]. The crescentic class (Berden classification) had the highest kidney function recovery capacity compared with other classes and the crescentic class is the predominant one in anti-GBM disease [17]. The latter group are mostly dialysis dependent at baseline and 79% of them had kidney failure at the end of follow-up. Crescents are important predictors of prognosis in AAV and anti-GBM disease consistently reported across studies, but their type and proportion vary across different populations [14, 18]. Japanese patients with ANCA-GN have cellular crescents in 63.8% of biopsies, with a mean of 11.2% cellular crescents, but only 5% of them were classified as belonging to the crescentic class [19]. This contrasts with studies in Caucasian populations, e.g. patients enrolled in four European Vasculitis Society (EUVAS) trials that had a mean proportion of cellular crescents of 40% with 45.3% of them belonging to the crescentic class [20].

Other GN forms that present with crescents, such as immunoglobulin A nephropathy (IgAN) and LN, are more heterogeneous. Patients with a high proportion of crescents are rarely included in clinical trials of IgAN and LN, as cGN may be a prespecified exclusion criterion and, even when they are included, outcomes of these patients are often not reported as a subanalysis [21, 22]. Cellular or fibrocellular crescents are present in 36% of IgAN patients, with 31% of these patients having >10% of crescents [23]. However, histology in IgAN differs across different populations, as active lesions, including crescents, are more common in Asian compared with European patients. For example, the Oxford classification study and subsequent validation studies showed that crescents were present in 63% of Japanese patients and 48% of Chinese patients but only in 17% of European cohorts [24–27].

The evolution of crescents was recently explored in LN, demonstrating similar findings as previously proposed in ANCA-associated GN. Cellular/fibrocellular crescents evolved to fibrous crescents at the second biopsy (a median of 9 months after the first), featuring only glomerulosclerosis by the third biopsy (median 34 months), suggesting that glomerulosclerosis is the endpoint of crescent evolvement, which might have important implications on potential for reversal [7, 28]. Data from CRESCENDO will help to further our understanding of reversibility and identifying the point of irreversibility.

There are notable differences in treatment regimens for AAV across Europe, Japan and the USA, as well as accessibility to treatments such as avacopan, which may influence patient outcomes. However, genetic, epigenetic, ethnic, environmental and other underlying factors could also contribute to these variations [29]. We will try to reduce this knowledge gap on efficacy and safety of therapy by including a global population and leveraging a TTE approach.

CRESCENDO will capture data on a broad set of outcomes, including not only death, KFRT and recovery from dialysis, but also major adverse cardiovascular events, severe infections and malignancy—all important when considering the aggressive immunosuppressive and cytotoxic therapy typically used in RPGN (Fig. 2).

Finally, CRESCENDO, as the largest global registry of cGN, will enable the design and execution of multiple important substudies, generating much-needed evidence currently lacking for most forms of cGN, such as dedicated studies on the impact of other histologic hallmarks, on the role of artificial intelligence and machine learning in the diagnosis and prognosis of cGN and on other potential biomarkers. The results of these studies will not only help to close existing gaps in cGN knowledge but also generate further hypotheses providing the basis for subsequent research.

## CRESCENDO INVESTIGATORS

The following CRESCENDO Investigators are authors of this article: Dwomoa Adu, Faten Aqeel, Federico Alberici, Salem Almaani, Urmila Anandh, Miha Arnol, Fatiu Arogundade, Ingeborg Bajema, Jonathan Barratt, Patricia Batković, Samira Bell, Anke von Bergwelt-Baildon, João Boavida, Jean Jacques Boffa, Peter Boor, Bhadran Bose, Olivia Boyer, Benoit Brilland, Jan Hinrich Bräsen, Silke R. Brix, Ivan Bubić, Stela Bulimbašić, David Buob, Martin Busch, Pablo Javier Cannata Ortiz, Fernando Caravaca-Fontan, Gordana Cavrić, Tiffany Caza, Aglaia Chalkia, Anand Chellappan, Chee Kay Cheung, Raquel Chorao, Angela Maria Cordoba Huerto, Marijana Ćorić, Sofia Correia, Zhao Cui, Ajay Dhaygude, Fatman Sule Kutlar Dursun, Ala Eldin Elhoweris, Emmanuel Esteve, Stanislas Faguer, Raul Fernandez Prado, Michael Fischereder, Jürgen Floege, Lauren Floyd, Lucy Francis, Eleni Frangou, Edouard Fu, Shouichi Fujimoto, Daniel P. Gale, Krešimir Galešić, Danica Galešić Ljubanović, Joana Gameiro, Lucia Cordero Garcia Galan, Duvuru Geetha, Loreto Gesualdo, Iolanda Godinho, Mario Gois, Ana Gomes, Dimitrios Goumenos, Muzamil Hassan, Marion Haubitz, Marc Hilhorst, Ivica Horvatić, Zdenka Hruskova,Tobias B. Huber, Jae Il Shin, Antonio Inacio, Wesam Ismail, Toshiko Ito-Ihara, Arenn Jauhal, David Jayne, Rachel Jones, Andreas Jonsson, Robert Kalyesubula, Talerngsak Kanjanabuch, Dino Kasumović, Jesper Kers, Nika Kojc, Alexandre Karras, Masao Kikuchi, Tamara Knežević, Thomas Knoop, Marek Kollar, Toshiyuki Komiya, Sradha Kotwal, Vojtěch Krátký, Vincenzo L’Imperio, Mario Laganović, Bärbel Lange-Sperandio, Ivo Laranjinha, Anna Levin, Sigrid Lundberg, Valerie Luyckx, Georgina Maalouf, Michele Mäske, Bojana Maksimović, Ana Malvar, Jolanta Malyszko, Sofia Marques, Bernardo Marques da Silva, Mansour Mbengue, Stephen McAdoo, Decsa Medika Hertanto, Juan M. Mejia Vilet, Luis Mendonca, Safak Mirioglu, Hitomi Miyata, Johann Morelle, Adam Morris, Rose Muhindo, Ritambhra Nada, Daiki Nakagomi, Karl Emil Nelveg-Kristensen, Brendon Neuen, Abdou Niang, Estela Nogueira, Sayna Norouzi, Balazs Odler, Sophie Ohlsson, Ikechi Okpechi, Jose Oliveira, Bolanle A. Omotoso, Louise Oni, Alberto Ortiz, Besa Osmani, Savas Ozturk, Marios Papasotiriou, Emma Pardo, Samir Parikh, Chen Au Peh, Anna Julie Peired, Marta Pereira, Nejc Piko, Manuel Praga, Dana Pramudya, Cedric Rafat, Raja Ramachandran, Manish Rathi, Thomas Rauen, Heather Reich, Ian Roberts, Katharina Rohner, Avi Rosenberg, Brad Rovin, Bjorn Runesson, Aleksandra Rymarz, David Saadoun, Aso Saeed, Mazdak Sanaei Nurmi, Beatriz Sanchez-Alamo, Jinny Sanchez-Rodriguez, Jan-Stephan Sanders, Sofia Santos, Ulf Schönermarck, Harald Seeger, Marten Segelmark, Claudia Seikrit, Haresh Selvaskandan, Tamer Shehab, Maria Jose Soler, Inga Soveri, Stefanie Steiger, Kate Stevens, Maria K. Svensson, Petar Šenjug, Martina Tedesco, Y.K. Onno Teng, Vladimir Tesar, Nicola M. Tomas, Jamie P. Traynor, Stephan Troyanov, Lisa Uchida, Martina Uzzo, Augusto Vaglio, Gisele Vajgel, George Vasquez-Rios, Željka Večerić Haler, Charlotte A te Velde-Keyzer, Matheus Vieira Goncalves, Angela Ye-Moon Wang, Maria Weiner, Maria A. C. Wester Trejo, Emelie Westergren, Thorsten Wiech, Julia Wijkstrom, Nikola Zagorec and Ming-hui Zhao.

Annette Bruchfeld, Andreas Kronbichler, Ingeborg Bajema, Peter Boor and Vanja Ivković are members of the Core Committee.

## Supplementary Material

sfag077_Supplemental_File

## Data Availability

Data from the registry will be made available upon reasonable request and approval of the Core Committee.

## Notes

The full list of CRESCENDO Investigators who are authors of this study are listed at the end of the article.
