# Supplementary material for: Building CRESCENDO – a global registry of glomerulonephritis with crescents
Source: Clin Kidney J. 2026 Mar 5;19(11):sfag077. doi: 10.1093/ckj/sfag077 (PMC13752502; doi:10.1093/ckj/sfag077)
Supplement: Supplementary file 1 [file sfag077_supplemental_file.docx]

**Supplementary material**

Outline of the Baseline and Follow-up Forms

Basic outline by data categories for each type of form is provided and specific data in each category is provided in Table 1.

*Baseline Form*

1. Background
2. Glomerulonephritis (GN) and organ involvement
3. Laboratory values
4. GN Scoring Form
5. Non-immunosuppressive therapy
6. Immunosuppressive therapy and plasma exchange (PLEX)
7. Outcomes

*Follow-up Forms*

Fixed Follow-up Form

1. Background
2. Organ involvement
3. Laboratory values
4. Non-immunosuppressive therapy
5. Immunosuppressive therapy and PLEX
6. New-onset comorbidities
7. Outcomes

Generic Repetitive Follow-up Form

1. Background
2. Organ involvement
3. Laboratory values
4. Non-immunosuppressive therapy
5. Immunosuppressive therapy and PLEX
6. New-onset comorbidities
7. Outcomes

Relapse and Re-biopsy Follow-up Form

1. Background
2. Glomerulonephritis (GN) and organ involvement
3. Laboratory values
4. GN Scoring Form
5. Non-immunosuppressive therapy
6. Immunosuppressive therapy and plasma exchange (PLEX)
7. New-onset comorbidities
8. Outcomes
